# Patterns in antipredator armature reduction and maintenance in isolated spring populations of an amphipod crustacean

**DOI:** 10.1002/ece3.10423

**Published:** 2023-08-29

**Authors:** John Loehr, Janne Sundell, Mikko Immonen, Risto Väinölä

**Affiliations:** ^1^ Faculty of Biological and Environmental Sciences, Lammi Biological Station University of Helsinki Lammi Finland; ^2^ Faculty of Biological and Environmental Sciences University of Helsinki Helsinki Finland; ^3^ Finnish Museum of Natural History Luomus University of Helsinki Helsinki Finland

**Keywords:** DNA, morphology, parallel evolution, predation

## Abstract

Organisms colonizing new habitats can undergo adaptive change due to novel selective landscapes encountered in the new environment. Examples in nature where the development of the same traits has repeatedly occurred on multiple independent occasions upon colonizing a novel habitat represent instances of parallel evolution. Here we test whether the colonization of spring habitat by the principally lacustrine amphipod crustacean *Pallaseopsis quadrispinosa* has resulted in parallel evolution in armature traits using empirical data on morphology and mitochondrial DNA and through a breeding experiment. Analysis of mtDNA CO1 sequences shows that the spring populations share no common history and have evolved in isolation from each other and from their neighbouring lake populations since deglaciation approximately 12,000 years ago and are now fixed for different haplogroups. Dorsal spines and lateral projections were absent or less developed in all spring populations than in lake populations. Variation in armature development also could be explained by predator presence as populations with fish predators exhibited more developed spines than those without fish. In a laboratory breeding experiment, hybrid Spring × Lake F1 offspring had intermediate development of armature compared to offspring of Lake × Lake and Spring × Spring matings. The results support the hypothesis that armature reduction has independently evolved on multiple occasions in *P. quadrispinosa*. Recent research has questioned the degree to which parallel evolution actually explains variance in traits. Taking into account the predation regime, sexual dimorphism and mineral composition of the trait, a more precise understanding of the factors influencing parallel evolution emerges.

## Graphical abstract

Using evidence from field sampling and laboratory experimentation, we describe how habitat differences and the presence or absence of fish predators influence spine morphology in an amphipod crustacean. Overall, such a clear and revealing demonstration of selection on an antipredator trait in a natural setting has great value for the understanding of the role of selection by predation on morphological evolution.

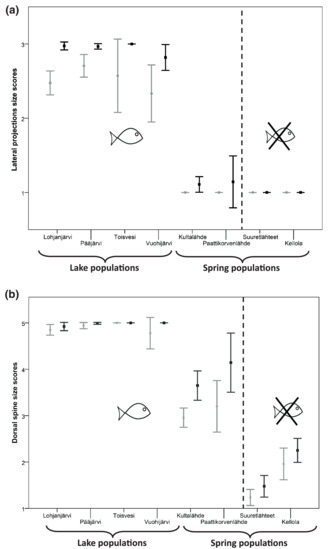

## 1 INTRODUCTION

Parallel evolution of morphological traits with independent occupancy of similar environments is generally interpreted as strong evidence of adaptation through natural selection and has been amply documented both at the interspecies and intraspecies level (e.g., Bolnick et al., 2018). Apart from parallel changes that reflect adaptation to a new selective regime by positive selection on new traits, parallel evolution may take place through the loss of presumably costly structures or functions when selection pressure for their maintenance is relaxed in a new environment (Lahti et al., 2009). The latter is well evidenced, for instance, in changes to a subterranean lifestyle in aquatic organisms, and examples are known, for example, from cave‐dwelling isopods (Mojaddidi et al., 2018) and amphipods (Jones et al., 1992).

A particularly well‐documented model of intra‐specific multiple parallel evolutions is that of the three‐spined stickleback *Gasterosteus aculeatus* (e.g. Schluter et al., 2004; Schluter & Nagel, 1995). Sticklebacks have repeatedly colonized different freshwater watersheds. On each occasion, their antipredator armature has gone through a reduction in genetically determined traits. Plausibly, in the freshwater environment, a reduced predation pressure has relaxed selection that maintains body armature enough to allow a reduction of the number of lateral bony armor plates. Freshwater individuals with fewer lateral plates grow faster than fully plated individuals, which could give the low plate morph a selective advantage when predators are few (Marchinko & Schluter, 2007). The genetic architecture of the traits has been identified and experimental evidence has also shown that the frequency of genes affecting armature in freshwater populations change over time (Barrett et al., 2008; Schluter et al., 2010). These examples of parallel evolution have been important in establishing and verifying natural selection as one of the primary drivers of diversification in the wild.

However, Oke et al. (2017) questioned to what degree parallel evolution can actually account for many of the purported instances of parallel evolution. They pointed out that in many cases, phenotypic variance between populations is high in the newly colonized environment making it difficult to know how much of the overall variance parallel evolution actually explains. Their analyses revealed that weak parallelism was frequent in a sample of published research on parallel evolution in fish. The variance in the predicted outcomes of colonization of similar habitats can result from an oversimplification of the categorization of habitats. For example, if ancestral versus colonized habitat is categorized based on saltwater populations colonizing freshwater habitats, there are numerous differences that can be present within the environment of each freshwater population that is not taken into account (Kaeuffer et al., 2012). Differing degrees of gene flow between ancestral and colonized habitats can also affect the degree to which selection affects the traits studied (Stuart et al., 2017). Thus, studies that can further quantify the actual differences between colonized habitats will help to elucidate selective pressures within the colonized environments and allow a better assessment of how much of overall variance parallel evolution can explain.

In the crustacean order Amphipoda, one example of intraspecies parallel evolution is from the species *Gammarus minus*, where cave‐dwelling populations have smaller eyes and larger antennae and body size than epigean spring populations (Jones et al., 1992). Copilaş‐Ciocianu et al. (2020) in turn surveyed Central European populations of *Gammarus roeselii* which has a dorsal carina with acute dentations, interpreted as an antipredator character. They found among‐population variation, which they posited to be a response to different predator regimes, but since predators were not known in the streams surveyed, and they did not determine whether spine growth was genetically determined or a plastic response to the environment, the cause of the variation is still unknown. Bollache et al. (2006) found that the presence of spines in *G. roeselii* aided this species to avoid predation in a microcosm experiment compared to a spineless species, *Gammarus pulex*. However, in this case, the comparison of a species that has spines to another that does not have spines limits interpretation. Thus, strong evidence combining laboratory breeding experiments and clear examples of multiple parallel evolution in the field is lacking leaving little concrete evidence to assess the specific evolutionary mechanisms that have led to the diversification within amphipods.

*Pallaseopsis quadrispinosa* is a ‘glacial relict’ freshwater species commonly found in deeper lakes of northeastern Europe and some coastal areas of the Baltic Sea (Segerstråle, 1957; Väinölä & Rockas, 1990). In this species, putative antipredator armature is present in four subdorsal spines on the pleon segments as well as in a row of lateral projections (‘supramarginal projections’) extending along the sides of the amphipod. The degree of development of these characters is known to vary between lake populations and examination of a peculiar spring population in southern Finland showed that armature is severely reduced (Segerstråle, 1958). While Segerstråle felt that the system presented an ‘unusually favourable opportunity for genetic experiments’, no further research has been undertaken. Since 1958, three more spring populations have been discovered in Finland, providing the opportunity to test whether the reduction of the armature has occurred in multiple populations and whether the traits have genetic or environmental underpinnings.

As with all North European fauna, the history of individual *P. quadrispinosa* populations is no older than the last deglaciation, c. 12,000 years (Stroeven et al., 2016). In general, the distribution of the “glacial relict” crustacean assemblage indicates that they cannot disperse upstream but are confined to areas once covered by initial (freshwater) stages of the Baltic Sea or earlier ice‐marginal waterbodies (Segerstråle, 1957; Väinölä & Rockas, 1990). The isolation times of populations can therefore be inferred from the history of hydrological connections, which is well understood from the reconstructions of the gradual post‐glacial land uplift, which caused the lakes and springs to become separated from the broader Baltic Sea basin, preventing further gene flow between drainages (e.g. Tikkanen & Oksanen, 2002).

In considering the evolution of the morphology, a first hypothesis is whether the changes took place independently in the different (spring) populations after their isolation, or whether the differences indeed would predate the colonization, and different habitat types were initially occupied by morphologically diverged strains. In the paleohydrological framework, this can be tested against independent genetic information on the genealogy of populations, if presumably neutral markers with sufficient resolution are available. The divergence of potential ancestral strains invading from outside the glaciated area would plausibly be traced back to the Last Glacial Maximum at least and thus be no less than twice older than the independent evolution in current waterbodies. Further, such molecular data could elucidate the demographic history of the independent populations, for the evaluation of a hypothesis that reduction of the armament could be a consequence of inbreeding, that is, effects or random loss of genetic variation in the small water bodies.

The other set of hypotheses relates to selection pressures and other ecological variables that plausibly could underlie the morphological differences. Particularly, since the spring and lake environments differ in their predator assemblages, the (repeated) reduction of armature in springs could have been facilitated by a relaxation of selection and a new selective regime. Also, differences in the strength of the armature could reflect the hydrochemical properties of the habitats.

Here we used multiple approaches to elucidate and test the patterns and causes of repeated reduction in body armature in the freshwater amphipod in waterbodies of different sizes and predator regimes, including qualitative morphometry, mitochondrial DNA variability and genealogy, interpopulation breeding experiments and environmental chemistry. The particular hypotheses tested with these data included:

The instances of reduced armature result from independent evolution in the isolated waterbodies, rather than representing old divergence dating back to times before the post‐glacial colonization of the region.

The reduction of armature is related to population size and could potentially reflect inbreeding history (loss of genetic variation).

If fish predators influence the maintenance of antipredator traits, populations from the spring and lake habitats should differ in antipredator armature in a consistent way with more developed armature existing in lake populations and reduced armature in spring populations.

During fieldwork we discovered that two of the springs contained fish predators, and we made a post‐hoc prediction that armature would be more developed in springs with fish.

The rigidity of crustacean exoskeletons is dependent on calcium carbonate (Nagasawa, 2012) and as an alternative hypothesis to H3 and H3b, the development of exoskeleton armature may depend on the availability of calcium. If armature development is limited by calcium availability, we expected that calcium would be found in lower concentration in water bodies with reduced armature and that amphipod exoskeletons with reduced armature would have a lower percentage of calcium ions than those with more developed armature.

If the armature trait is heritable, the F1 generation of common garden, laboratory‐raised amphipods from springs and lakes are morphologically similar to their parent populations.

## 2 METHODS

### 2.1 Study species and habitat

*Pallaseopsis quadrispinosa* is a primarily benthic, omnivorous, sexually dimorphic freshwater amphipod. Males can be up to 2.5 cm in length and females are approximately 30% smaller. Life span is 1–2 years; reproduction can occur throughout the year, but most commonly in autumn and winter (Hill, 1988). The species is commonly found in relatively deep lakes of Northern Europe where cold water is available year‐round (Segerstråle, 1957). This species exhibits body armature of the exoskeleton, most notably in the occurrence of two pairs of subdorsal spines on pleon segments 1 and 2, and a row of lateral projections (‘knobs’) on the pereon segments. The degree of development of the armature is known to vary between lake populations (Segerstråle, 1958). Particularly, a study of the first identified spring population (Suuretlähteet) revealed severely reduced armature with practically no dorsal spines (Segerstråle, 1958).

*Pallaseopsis quadrispinosa* is preyed upon by fishes. For instance, in Lake Pääjärvi, it has been found in stomach of burbot, pikeperch, ruffe and perch (Kimmo Kahilainen, personal communication), while the whole spectrum of predators is not known. During the study, we also observed that the brown trout *Salmo trutta* ingested *P. quadrispinosa*. Invertebrate predators probably include diving beetles and Odonata larvae. In several of the deepest Finnish lakes, *P. quadrispinosa* also coexists with the predatory amphipod *Gammaracanthus lacustris*. However, from numerous sampling campaigns in Lake Pääjärvi, we have not observed that *G. lacustris* predates on *P. quadrispinosa* when kept in small 4 L containers post‐sampling, although it readily predates on *Mysis relicta*.

**FIGURE 1:**
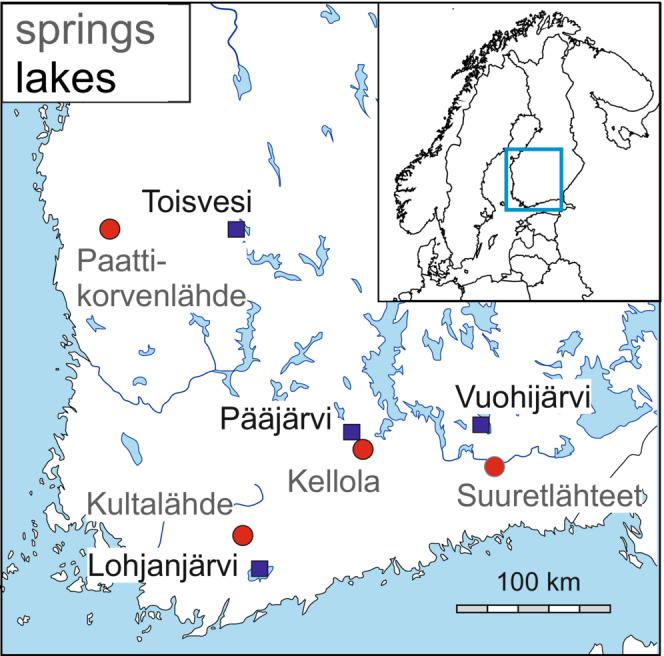
Locations of springs and lakes in southern Finland from which *Pallaseopsis quadrispinosa* were sampled. Fish predators were absent from the Kellola and Suuretlähteet Springs, but their presence was confirmed in Kultälähde and Paattikorvenlähde Springs. Base map(c) https://d‐maps.com/.

Apart from the Suuretlähteet Spring studied by Segerstråle (1958), *P. quadrispinosa* is now known from three other springs in Finland (Figure 1). Predatory diving beetles (*Dytiscus* spp.) have been observed in the springs and it is likely that other invertebrate predators are also present. During the fieldwork, we assessed predator presence visually in spring population and found that two of the four springs‐contained fish predators. Northern pike (*Esox lucius*) was observed in Paattikorvenlähde Spring and brown trout (*Salmo trutta*) was found in the outlet stream. *S. trutta* was also present in Kultalähde Spring. These two springs differ from the fishless springs in that their outlet streams drain directly into the Baltic Sea with a direct colonization route for brown trout to the spring. These streams are marked as potentially trout‐inhabited streams by the Ministry of Agriculture and Forestry (see https://kalastusrajoitus.fi/). The other two springs are separated from larger streams (possibly trout inhabited) by greater distances and their small outlets are not marked as possible trout streams.

### 2.2 Sampling

**FIGURE 2:**
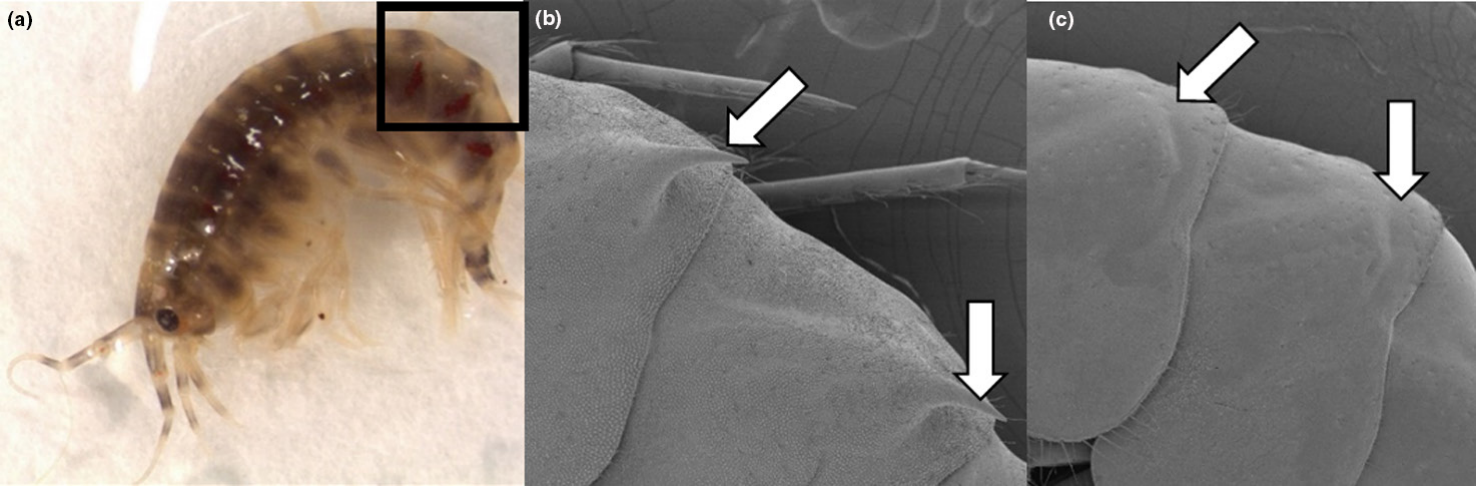
(a) Photograph of adult female *Pallaseopsis quadrispinosa* from Kellola Spring population, length about 12 mm. Black rectangle indicates the area that has been enlarged in panels b and c. (b) Scanning electron microscope image shows the subdorsal spines on pleon segments 1 and 2 as indicated by arrows, from the Lake Pääjärvi population and (c) a Suuretlähteet Spring individual with no spines. Arrows point to areas where spines are absent.

Sampling was carried out in the four springs known to be inhabited by *P. quadrispinosa* and the closest large lake to each of those springs (Figure 2, Table 1). The springs are ponds whose surface areas range from 200 to 400 m^2^ and depths from 4 to 8 m and which have year‐round water temperatures of ca. 6°C. Lake surface areas range from 13.4 to 88.2 km^2^ and maximum depths 55–85 m.

**TABLE 1:** The eight spring and lake populations of *Pallaseopsis quadrispinosa* studied, with habitat classification, the presence of fish predators and the numbers of individual males and females used in the analysis of morphological traits.

Population	Habitat	Fish predators?	Coordinates (WGS84)	Altitude (m)	Males	Females
Kellola	Spring	No	61°00'33" N, 25°11'53" E	96	20	24
Pääjärvi	Lake	Yes	61°3'57" N, 25°8'16" E	103	92	51
Kultalähde	Spring	Yes	60°27'27" N, 23°37'39" E	82	37	46
Lohjanjärvi	Lake	Yes	60°14'33" N, 23°58'12" E	32	38	40
Suuretlähteet	Spring	No	60°54'51" N, 27°02'14" E	68	21	34
Vuohijärvi	Lake	Yes	61°10'37" N, 26°42'08" E	77	22	9
Paattikorvenlähde	Spring	Yes	62°10'26" N, 022°02'43" E	109	7	5
Toisvesi	Lake	Yes	62°17'52" N, 23°43'17" E	98	4	7

Adult *P. quadrispinosa* were captured using dip nets (spring populations), a baited plankton net (Lake Pääjärvi), or benthic sledge (other lake populations). Sampling for breeding experiments was performed between September and October 2013, and for morphological analysis between September and November 2014, except for Lake Toisvesi which was sampled in August 1986. Specimens taken for morphological analysis were placed into a −80°C freezer within 24 h of sampling. The number of males and females sampled, habitat type and the presence/absence of fish predators are listed in Table 1. Samples for DNA analysis were taken from both the 2013 and 2014 fieldwork and from previously deep‐frozen material (Väinölä & Rockas, 1990). In total, we sampled 98 individuals from springs with no fish (40 males and 58 females), 135 individuals from springs with fish (44 males and 91 females) and 263 individuals from lakes (156 males and 107 females).

### 2.3 Scoring of sex and morphology

Sex of individuals was determined by observing precopulatory breeding pairs (males hold females for 1–3 days before copulation) and the presence of a post‐copulatory marsupium in females. The species is sexually dimorphic and the largest of the individuals from any given population were males. We also noted that adult males could be distinguished by their swimming posture as the first 3–4 segments of the pereon behind the head aligned in a straight line (when viewed laterally). In contrast, the majority of adult females appear smoothly curved for the entire length of their body; viewed dorsally their body is noticeably widened around the 2–5 pereon segments. This trait is not always visible in females, which may be due to it developing with the development of the marsupium and being retained for some time afterwards.

The armature development of typical lake and spring individuals is presented in Figure 2 and in Appendix A. Body armature was assessed visually using a stereomicroscope with 10–30× magnification. The degree of armature was scored subjectively, for spines on a scale of five categories (1—not visible or very small to 5—fully present), and for the lateral projections in three categories. Reference figures for the scoring (Appendix A) were made based on diagrams from Segerstråle (1958). The repeatability of the ranking method was verified in a test where two biology students scored 30 individuals from two spring and two lake populations (Cronbach's alpha, α = 0.98 for the spines, α = 0.90 for the lateral projections, IBM SPSS Statistics v 22). Body length was estimated by placing individuals on graphing paper and measuring the distance along the dorsal line from the base of the telson to the tip of the cephalon in a stretched position (Nahavandi et al., 2011).

### 2.4 Breeding experiment

A breeding experiment was performed in which individuals from lake and two spring populations were placed in population‐specific 40 L aquaria kept at 6°C with a 12:12 h light–dark cycle. Water was changed at approximately 1‐month intervals. The aquaria were filled with untreated tap water which is sourced from groundwater. Nourishment for offspring was provided by placing dry *Populus tremula* leaves from the forest floor on the bottom of the aquarium. Offspring were occasionally provided with small amounts of aquarium fish food flakes as a supplement to their diet.

For within‐population breeding, individuals were allowed to mate within their aquaria, which contained wild captured (September/October 2013) individuals from Lake Pääjärvi (*n* = 191), Kellola Spring (*n* = 77), and Suuretlähteet Spring (*n* = 25). On 2 December 2013, females (Lake Pääjärvi *n* = 20; Kellola Spring *n* = 20) carrying fertilized eggs were removed from their breeding aquaria and placed in two separate population‐specific aquaria to bear offspring. The small number of individuals from Suuretlähteet spring were left to breed in their own aquarium. The first offspring were discovered on 2 January 2014.

Using wild‐captured individuals from September/October sampling, Lake × Spring hybrid offspring were produced. On 20 January 2014, 25 Lake Pääjärvi females were added to an aquarium containing >20 Kellola Spring males. In a separate aquarium, 22 Kellola Spring females were added to an aquarium with >20 Lake Pääjärvi males. Prior to placement in male aquaria, all females were observed under a microscope to ensure that they were not carrying offspring or eggs.

In all aquaria, once offspring were produced, adult males and/or females were removed to minimize the risk of cannibalism. On 22 May 2014, to save space the number of aquaria was reduced to three. One aquarium held the Lake Pääjärvi offspring, a second held offspring from the spring habitat populations, and a third held all offspring of the Lake Pääjärvi and Kellola Spring crosses.

The breeding experiment was terminated on 9 January 2015. Morphological characteristics of all surviving offspring were recorded before they were placed at −80°C. In total, 18 Spring × Spring, 6 Spring × Lake and 2 Lake × Lake offspring were found. Individual length at this time was 4–11 mm, which was equal to or greater than the 4 mm of length observed for full armature growth in wild populations (Segerstråle, 1958). At this length, however, individuals were too small to be sexed with confidence.

### 2.5 Calcium ion analyses

Water samples for calcium ion analysis were taken using a 2 l Limnos sampler from lakes and with a 1 l plastic bottle from springs. Thawed *P. quadrispinosa* individuals (*n* = 118) were dried at 60°C for 24 h, then weighed and incinerated in a Carbolite ESF3 furnace (Carbolite, Hope Valley, UK) at 550°C for 1 h. The inorganic ash was then weighed and compared with the previously measured dried mass. The ash and water mineral concentrations were analysed in a Varian SpectrAA 220FS spectrometer. Measurements were made only on males to avoid the possibly confounding effects of female reproductive status on results (Glazier et al., 2003). Individuals that had recently moulted and had soft exoskeletons (*n* = 2) were excluded from the analysis.

### 2.6 Statistical analysis

We analysed the morphological differences between lake and spring habitats and fish predator presence or absence using general linear mixed models, as implemented in IBM SPSS Statistics v 22. The scores of dorsal spines and lateral projections were entered as dependent variables using a multinomial distribution to take into account the ordinal scale scores of the dependent variable. Sex, individual length habitat type, fish predator presence, and the interaction between sex and habitat type and sex and fish presence were entered as independent variables. For the model assessing lateral projection development, the interactions were not significant and were not considered in the final model. The population was fitted as a random factor. Our sample sizes were unbalanced and we used the Saitherthwaite approximation to estimate degrees of freedom. Exoskeleton content analysis was analysed using a *t*‐test, and morphological differences of offspring from the breeding experiment were analysed using non‐parametric Kruskal–Wallis tests and Mann–Whitney *U* tests for post‐hoc multiple comparisons between groups using IBM SPSS Statistics v 22.

### 2.7 Mitochondrial DNA analysis

DNA was isolated from pleopods of individual amphipods which had been stored either deep‐frozen or in 96% ethanol, using a salt‐extraction protocol (Aljanabi, 1997). A fragment of the mitochondrial COI gene was amplified and sequenced using the standard barcoding primers LCO and HCO (e.g. Laakkonen et al., 2021: Appendix A). Sequences were obtained from *N =* 12 individuals from each of the populations except the Kultalähde Spring (*N =* 11; Table 2). [Data were deposited in GenBank under accession numbers OR198382–OR198477].

**TABLE 2:** Statistics of mitochondrial DNA variation of the COI gene within and among *P. quadrispinosa* populations.

Population		*N*	*Nh*	π (%)	Net distance(%)	φ_ST_
Kellola*	Spring	12	4	0.17		
Pääjärvi	Lake	12	5	0.16		
Kultalähde	Spring	11	4	0.20		
Lohjanjärvi	Lake	12	3	0.13		
Suuretlähteet*	Spring	12	2	0.03		
Vuohijärvi	Lake	12	3	0.18		
Paattikorvenlähde	Spring	12	3	0.05		
Toisvesi	Lake	12	2	0.03		
Springs		(47)	(13)	0.11	0.73	0.86
Lakes		(48)	(13)	0.12	0.43	0.78
Total		(95)	(26)	0.12	0.49	0.80

*Note*: *N –* sample size, *nh –* number of different haplotypes observed, π (%), intrapopulation nucleotide diversity (average percentage of nucleotides different between individuals), net distance: average interpopulation divergence (corrected for intrapopulation diversity), φ_ST_ – proportion of interpopulation nucleotide diversity proportion. The values in parentheses are total for the pooled material, others are mean values. Asterisks denote fishless springs.

From an alignment of 623 bp, a TCS haplotype network and maximum parsimony trees were generated using PopART 1.7 (Leigh & Bryant, 2015) and MEGA X (Kumar et al., 2018). A sequence from a White Sea (Arctic) drainage population was included as an outgroup for the ingroup Baltic Sea drainage populations in these analyses. Molecular diversity and distance statistics were obtained with MEGA X and DnaSP 5.0 (Librado & Rozas, 2009).

## 3 RESULTS

### 3.1 Mitochondrial DNA variation (H1 and H2)

**FIGURE 3:**
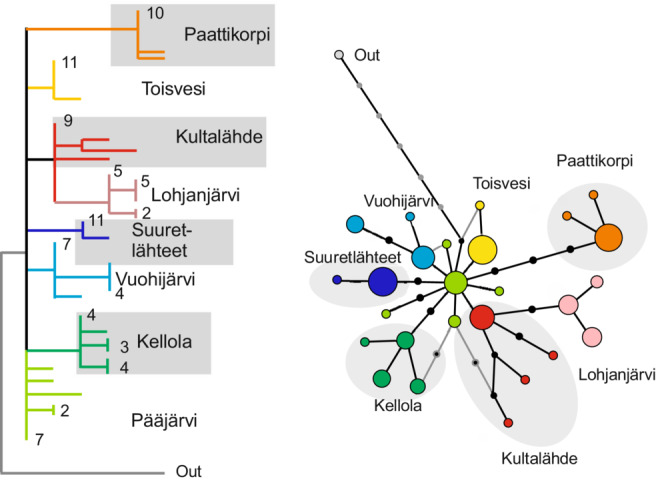
Genealogy of mitochondrial DNA from lake and spring populations, inferred from 623‐bp COI gene sequence. (a) MP parsimony tree (one of the nine equally parsimonious MP solutions) (b) TCS haplotype network. Springs are indicated with background shading. The number of multiple identical haplotypes is indicated by numerals in a and by the circle area in b. The outgroup sequence is from Lake Paanajärvi in the White Sea drainage basin.

In the inferred mitochondrial genealogy, almost all populations made separate, non‐overlapping clusters or clades. The Lake Pääjärvi population however appeared in a basal position in the parsimony tree and statistical parsimony network (Figure 3). The interrelationships among individual populations were mostly unresolved. Particularly, the different spring populations did not cluster together. The geographically adjacent spring versus lake population pairs generally did not group together, apart from the Lake Lohjanjärvi‐Kultalähde Spring pair. The spring population did not appear as being derived from the lake population, but rather vice versa in accord with the lake being the younger population in this case.

In terms of nucleotide diversity, the total variability in the data was π_T_ = 0.61% (i.e. average individuals differed by c. 3.7 nucleotide sites in the studied 623 bp fragment). Most of this variation (φ_ST_ = 80%) was between populations and only 20% within populations (average intrapopulation diversity π_S_ = 0.12%). The net interpopulation differences were c. 70% greater among spring populations (0.73%) than among lakes (0.43%), whereas pairwise lake‐spring distances (0.50%) were similar to the overall mean (0.49%) (Table 2).

Samples from all individual waterbodies showed some intrapopulation variation. The number of observed haplotypes was 2–5 per population and estimates of nucleotide diversity 0.03%–0.20%. The average diversity in spring and lake populations was similar (π = 0.11%, 2–4 haplotypes vs. π = 0.12%, 2–5 haplotypes). Nevertheless, two of the four springs were almost monomorphic, with only 1–2 deviating haplotypes, but so was one of the lakes Lake Toisvesi.

### 3.2 Variation in body armature (H3 and H3b)

**FIGURE 4:**
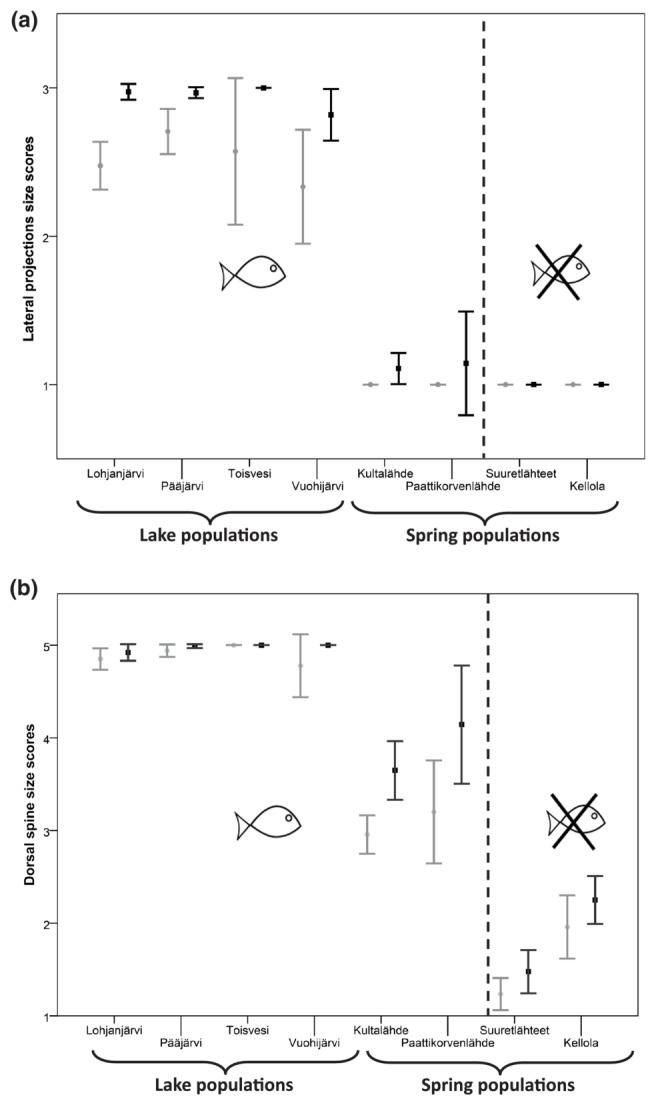
95% confidence intervals for the means of antipredator armature development scores for (a) lateral projections (ordinal scale of 1–3) and (b) dorsal spines (ordinal scale of 1–5) in lake and spring populations of the amphipod *Pallaseopsis quadrispinosa*. Higher scores indicate greater developed armature. The dotted line separates those populations that have fish predators on the left from the two populations on the right that do not have fish. The grey error bars represent females, and the black error bars represent males.

GLMM analyses of armature trait scores revealed that both dorsal spines and lateral projections were more developed in lakes than in springs (H3) Lateral projections were more developed in males than in females, and the same pattern was evident in dorsal spines but only when springs were analyzed separately (Table 3, Figure 4). Spines were more developed in populations with fish than those without, but no effect of fish was found on the lateral projections. The interactions between habitat and sex were not significant for spines and lateral projections, while the interaction between fish presence and sex was only significant for dorsal spines (Table 3, Figure 4). To test H3b, we constructed a second GLMM with only spring populations included (Table 4). We found a non‐significant trend for more developed spines in populations with fish compared to those without fish and spines were more developed in males than in females.

**TABLE 3:** General linear mixed model of variables predicting the amphipod armature trait development (dorsal spines and lateral projection scores) for both spring and lake populations.

Variable	Dorsal spines				Lateral projections			
	df_1_	df_2_	*F*	*p*	df_1_	df_2_	*F*	*p*
Corrected model	5	23	16.8	<.001	4	104	21	<.001
Fixed effects								
Sex	1	447	3.2	.073	1	451	34.8	<.001
Habitat	1	6	29.7	.001	1	26	66.5	<.001
Fish presence	1	5	15.6	.012	1	451	0	.986
Body length	1	447	2.7	.099	1	451	1.5	.217
Sex × habitat	1	447	1.4	.235				
Sex × fish presence	1	447	5.5	.019				

Random effects	Est.	SE	Z	*p*	Est.	SE	Z	*p*
Population	1.1	0.9	1.28	.202	0.3	0.4	0.89	.372

Abbreviations: Est., Estimate; SE, Standard error.

**TABLE 4:** General linear mixed model of variables predicting the amphipod dorsal spine armature development scores for spring populations only.

Variable	Dorsal spines			
	df_1_	df_2_	*F*	*p*
Corrected model	3	2	12.2	.053
Fixed effects				
Sex	1	186	5.3	.022
Fish presence	1	2	8.5	.092
Body length	1	186	7.2	.008
Sex × fish presence	1	186	5.7	.018

Random effects	Est.	SE	Z	*p*
Population	2.4	2.5	0.94	.347

Abbreviations: Est., Estimate; SE, Standard error.

### 3.3 Calcium ion analysis (H4)

Calcium ions made up a greater percentage of male exoskeleton ash in spring individuals compared to lake individuals (*t* test, *t* = −2.85, df = 116, *p* = .005) indicating that the rigidity of the exoskeleton was greater in spring populations. This trend does not appear to exist due to calcium availability in the water of each population as the mean calcium concentration for springs (x = 7.5 ± 3.4 μg/L) and lakes (x = 7.5 ± 2.7 μg/L) was similar.

### 3.4 Breeding experiment (H5)

**FIGURE 5:**
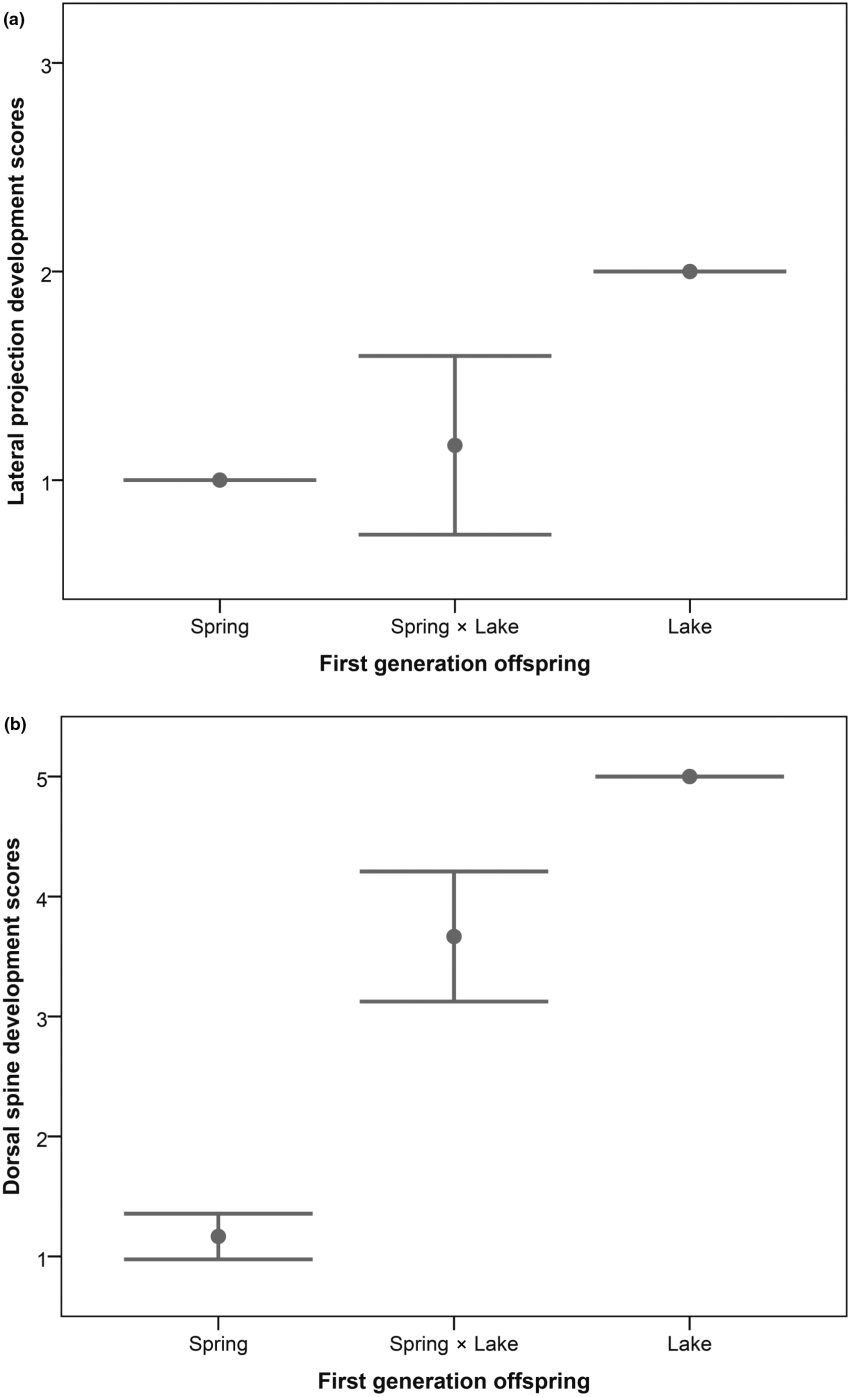
Mean antipredator armature development scores (with 95% confidence intervals) in F1 offspring in the common garden experiment. (a) *Lateral projection development scores (scale 1–3, see Appendix A) dorsal spine development scores (scale 1–5). Sample sizes Spring × Spring *n* = 18, Lake × Spring (*n* = 6) and Lake × Lake crosses (*n* = 2).

F1 offspring from spring, lake and hybrid populations differed significantly in their lateral projection development scores (Kruskal–Wallis test, H = 17.2, df = 2 *p* < .001, Figure 5a) and dorsal spines (Kruskal–Wallis test, H = 20.4, df = 2, *p* < .001, Figure 5b). Post‐hoc multiple comparisons using Mann–Whitney *U* tests revealed significant differences (*p* ≤ .05) between all offspring groups except in lateral projections between the Spring × Spring versus hybrid population comparison (*p* = .083). Since these traits were found to be sexually dimorphic and we were not able to assess the sex of the juvenile offspring from the breeding experiment, there is a possibility that results are also influenced by sex, but it is unlikely to affect the overall conclusions of the analysis.

## 4 DISCUSSION

The molecular evidence that the spring populations share no common precolonization history that could account for their morphological congruence was very clear. Such a shared history would be reflected as separate spring and lake lineages that would appear considerably deeper than the inferred post‐glacial divergence among individual springs (or lakes). The data instead display a polytomy that points to independent radiation of all populations from a common post‐glacially colonizing *P. quadrispinosa* stock, with no observed haplotype sharing among populations in the data. Most populations, both springs and lakes, comprise separate, clearly monophyletic branches in the mitochondrial tree. Nevertheless, the dominant haplotype in the Pääjärvi population is shared widely with other populations in the Baltic Sea basin (R. Väinölä, unpubl. data) and is inferred to have been common at the time of colonization, c. 12,000 years ago. Overall, the result supports our Hypothesis H1, that reduction of body armature took place independently in the post‐glacially isolated spring populations.

Another insight from the molecular data is that the spring populations on average show almost as much intrapopulation diversity (*π*) as the lakes. As this diversity component is governed by the (post‐isolation) population size, there is no support for an idea (H2) that the reduced armature would be generally attributable to a severe inbreeding history in small populations. While two of the four spring populations indeed had very little mitochondrial variation, there was no obvious covariation between this variation and armature size in the spring populations. We note that our estimates of diversity only represent the mitochondrial genome, effectively a single gene; their extension to broader genetic variation depends on the prediction that effects of demography are similar for all parts of the genome.

The apparent intrapopulation mitochondrial monophyly along with the measurable levels of intrapopulation diversity allow some further conclusions of population demography and of molecular evolution. The pattern *P. quadrispinosa* is quite similar to that reported earlier from Swedish lake populations of another ‘glacial relict’ crustacean species *Mysis salemaai*, where mtDNA of populations likewise appear as monophyletic clusters (Audzijonyte & Väinölä, 2006), and we can here follow the argument in that study. From geological history (Baltic shoreline displacement) and the current elevations (Table 1), the population ages are in the range of 6–11 ky, and for convenience, we will use *t* = 10 ky as an operational average. The time in generations would be about the same. First, in our framework, the interpopulation (net) distances (average d = 0.49%) would serve as estimates of mutational distances since the radiation and imply an mtDNA divergence rate of ~0.5% /10 ky, similar to or even higher than for the *Mysis* data of Audzijonyte and Väinölä (2006) (0.27%/10 ky). Second, the expected time to the coalescence of the mitochondrial population (i.e. monophyly) is 2*N*_ef_ generations, and populations smaller than *N*_ef_ < t/2 (~5000) are needed to account for the coalescence inferred to have taken place within each population (Hudson, 1990). Moreover, in our data, the intrapopulation coalescences indeed are notably more shallow than the interpopulation divergence assumed as 10 ky—using the mean distances or diversities of Table 2 as a guide only ca. 25% of them (d = 0.12%), and thus potentially only c. 2.5 ky old, implying *N*_eF_ < 1250 over that period. These estimates are rough and should only serve as approximations, to the order of magnitude. The magnitudes, however, notably deviate from conventional expectations, which would involve slower rates and plausibly larger population sizes for the lakes at least. On the other hand, the inferences of independent population history would not have been possible under the more conventional molecular rates.

We found support for the idea that populations from lakes had more developed armature than those from spring habitats (H3) and partial support for the hypothesis that springs with fish had more developed armature than those without fish (H3b). The full model supported the idea that populations with fish have more developed spines, however, a second model assessing only spring populations gave only weak statistical support for this trend. We could reject H4 as there was no indication that the calcium concentration of waterbodies or the concentration of calcium ions in amphipod exoskeletons is correlated with armature traits. Finally, we found support for the idea (H5) that purebred and hybrid offspring from common garden experiments would resemble their parents suggesting a genetic basis for the armature traits.

Overall, evidence suggests that dorsal spines are advantageous as an adaptation to fish predation. Armature reduction in the spring populations may be the result of differences in the predator regimes. These environments were correlated with the morphology and variance in dorsal spines, however, the influence of fish predation on lateral projections was not evident. Presumably, the spines of *P. quadrispinosa* are beneficial by increasing predator gape size necessary to swallow prey. In populations with fish presence, the effect of spines may be especially relevant as amphipods may gain sufficient protection from more developed spines when fish predators attempt to ingest them. The backward sweeping spines may make swallowing of amphipods difficult if the spines catch on the mouth or esophagus.

The absence of fish predators in two of the springs has resulted in an environment where selection by fish predation is relaxed. As outlined by Lahti et al. (2009), relaxed selection can influence traits through three main avenues: 1. Direct fitness benefits from spines protecting from fish predation are no longer incurred, however, costs may be incurred from the spines if grasping of spines by invertebrate predators is more successful. For example, dragonfly larvae *Leuchorrinia caudalis* with experimentally reduced spines suffered lower predation by aeshnid predators (Mikolajewski et al., 2006), although these same spines are advantageous to the prey when confronted with fish predation. 2. Indirect costs from spines may be incurred if the trait is genetically or structurally correlated with other traits. For example, if burst speed is hindered by increased drag incurred by the antipredator armature, this may result in spines having a disadvantage under a new predator regime where the spines are not beneficial. Spines and projections could experience reduction over time in the event that there is a trade‐off between growth rate and armature formation and higher growth rates benefitted fitness in an environment with no fish predators. This trade‐off between growth and armature is evident in three‐spined sticklebacks in freshwater habitats in which those with reduced plate numbers have more rapid juvenile growth than fully plated individuals (Marchinko & Schluter, 2007). 3. Finally, neutral mutations can influence the evolution of non‐functional traits and their decay over time, although, in this case, we would not expect to see a strong correlation between the environment and morphology as is evident in *P. quadrispinosa* populations. Similarly, founder effects and genetic drift could result in the loss of traits, but in this case, we would not expect to find a correlation between environment and morphology.

The selective landscapes present in each of the three environments likely have some bearing on the variance in traits found in the populations. Most clear is the situation in lake populations where there was very little dorsal spine variance suggesting that high selection pressure is imposed by fish and selects against individuals with less developed spines. Both spring environments (with and without fish) had comparatively greater variance in spines. Possible reasons for this include opposing selection pressures in springs with fish, where invertebrate and piscine predation select on the trait in opposite directions, but in a manner more balanced than in lake populations where selection from fish predation may be strongest. Variance may also be a result of relaxed selection where selection is no longer strongly imposed on the trait and small variations in the trait are not strongly relevant to fitness outcomes. In the case of springs with fish, the amount of time that has elapsed since fish have been present may also be relevant. Since we do not know how long fish have been present in the springs it is possible that a more recent arrival of fish predators is resulting in a gradual change in the spine trait (that is now intermediate between lake and spring without fish populations) that will eventually lead to the trait resembling that of lake populations. However, the fact that the two springs with fish are directly connected to the Baltic Sea and known trout streams suggest that the presence of fish has been long term.

Lateral projections were sexually dimorphic, with more developed armature in males, and in spring populations, dorsal spines were more developed in males. Sex differences in morphology may simply be a result of pleiotropic effects of other sex‐linked traits under selection, however, this trend may also develop if there is greater selection pressure by predators on males. In this way, antipredator armature could have greater importance in *P. quadrispinosa* males if they have higher activity levels that expose them to predation more. Higher activity levels could possibly arise from searching for mates or supporting their larger body size by more time spent foraging. Activity budgets of the amphipod *G. pulex* gathered in a laboratory setting indeed showed higher activity in males (Peeters et al., 2009), however, Lipkowski et al. (2019) found the opposite to be true in *G. roeselii*.

In conclusion, our genetic and morphological results demonstrated that reduction of antipredator armature has occurred repeatedly when populations have been isolated in spring habitats. This morphological change appears to have a genetic basis as first‐generation laboratory‐bred offspring resemble their parents. We found an overall effect of the presence of fish predators on the development of spines and a tendency for spring populations without fish to have less developed spines than those with fish. Overall, the *P. quadrispinosa* system shows great potential to better elucidate the evolutionary factors involved in shaping the outcome of parallel evolution.

## AUTHOR CONTRIBUTIONS

**John Loehr:** Conceptualization (equal); data curation (lead); formal analysis (lead); investigation (lead); methodology (lead); resources (equal); supervision (equal); writing – original draft (lead); writing – review and editing (lead). **Janne Sundell:** Conceptualization (equal); methodology (equal); resources (equal); supervision (equal); writing – review and editing (equal). **Mikko Immonen:** Conceptualization (equal); investigation (equal); methodology (lead); writing – review and editing (supporting). **Risto Vainola:** Conceptualization (equal); data curation (lead); formal analysis (lead); investigation (lead); methodology (lead); writing – review and editing (equal).

## FUNDING INFORMATION

Unfortunately, none of recieved funding directly for this work. It was all done as a side project.

## Data Availability

The data that support the findings of this study will be made openly available in Dryad at DOI: 10.5061/dryad.7m0cfxq15, reference number (ECE‐2023‐01‐00094.R2) and Genbank, reference number (OR198382–OR198477).
